# Doyne honeycomb retinal dystrophy/malattia leventinese induced by *EFEMP1* mutation in a Chinese family

**DOI:** 10.1186/s12886-018-0988-7

**Published:** 2018-12-12

**Authors:** Kaiyan Zhang, Xuyang Sun, Yingying Chen, Qionglei Zhong, Lin Lin, Yuan Gao, Fanlin Hong

**Affiliations:** Department of Ophthalmology, Hainan General Hospital, Haikou, 570102 China

**Keywords:** Doyne honeycomb retinal dystrophy, Malattia leventinese, Drusen, Visual acuity

## Abstract

**Background:**

Doyne honeycomb retinal dystrophy (DHRD)/malattia leventinese (ML) is a rare allelic condition with massive drusen in the posterior fundus caused by *EFEMP1* gene mutation. Patients showed decreased vision when the lesion affected the macular area. At present, the treatment efficiency is not satisfactory.

**Case presentation:**

In this study, we presented a family with DHRD/ML disease and analyzed the pathological and genetic information. A 28-year-old female patient presented to our department due to impaired visual acuity for 10 years especially in the right eye with deterioration for 5 months. Gene sequencing was performed by MyGenostics (Peking, China). Gene sequencing results revealed heterozygous mutations in *EFEMP1* gene, which were consistent with the DHRD/ ML. Single heterozygous mutation (*c.1033C > T*) was observed in each of the three blood samples. This missense mutation triggered p.R345W.

**Conclusions:**

DHRD/ML is a rare disease associated with *EFEMP1* gene mutation. Up to now, we are not sure whether these lesions are associated with the onset of DHRD/ML. In future, we hope to find out the exact relationship between them.

## Background

Doyne honeycomb retinal dystrophy (DHRD)/Malattia leventinese (ML) is caused by a single mutation in *EFEMP1* that encodes the epidermal growth factor-containing fibrillin-like extracellular matrix protein 1 [1]. In its early stages, the disease appears as massive drusens, while the visual acuity of the patients would decrease with the lesion progress [2]. To date, laser therapy and anti-VEGF therapy have been utilized for the treatment, but the treatment is still a challenge as there is no effective way to correct the gene mutation of such condition [3]. In this study, we reported a family with DHRD/ML disease that presented different features with the former reports. It contributed to the diversity of DHRD/ML.

## Case presentation

**Fig. 1:**
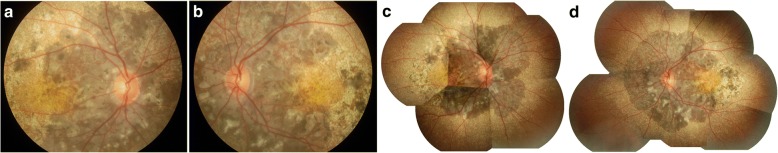
Fundus color photography. RPE/choroid atrophy was noticed in the posterior fundus around the disc, with diffuse pinpoint or speckle yellowish white lesions involving the peripheral area reached to the equator (**a**: right eye; **b**: left eye). Color photo puzzle showed relatively normal findings in peripheral fundus (**c**: right eye; **d**: left eye)

**Fig. 2:**
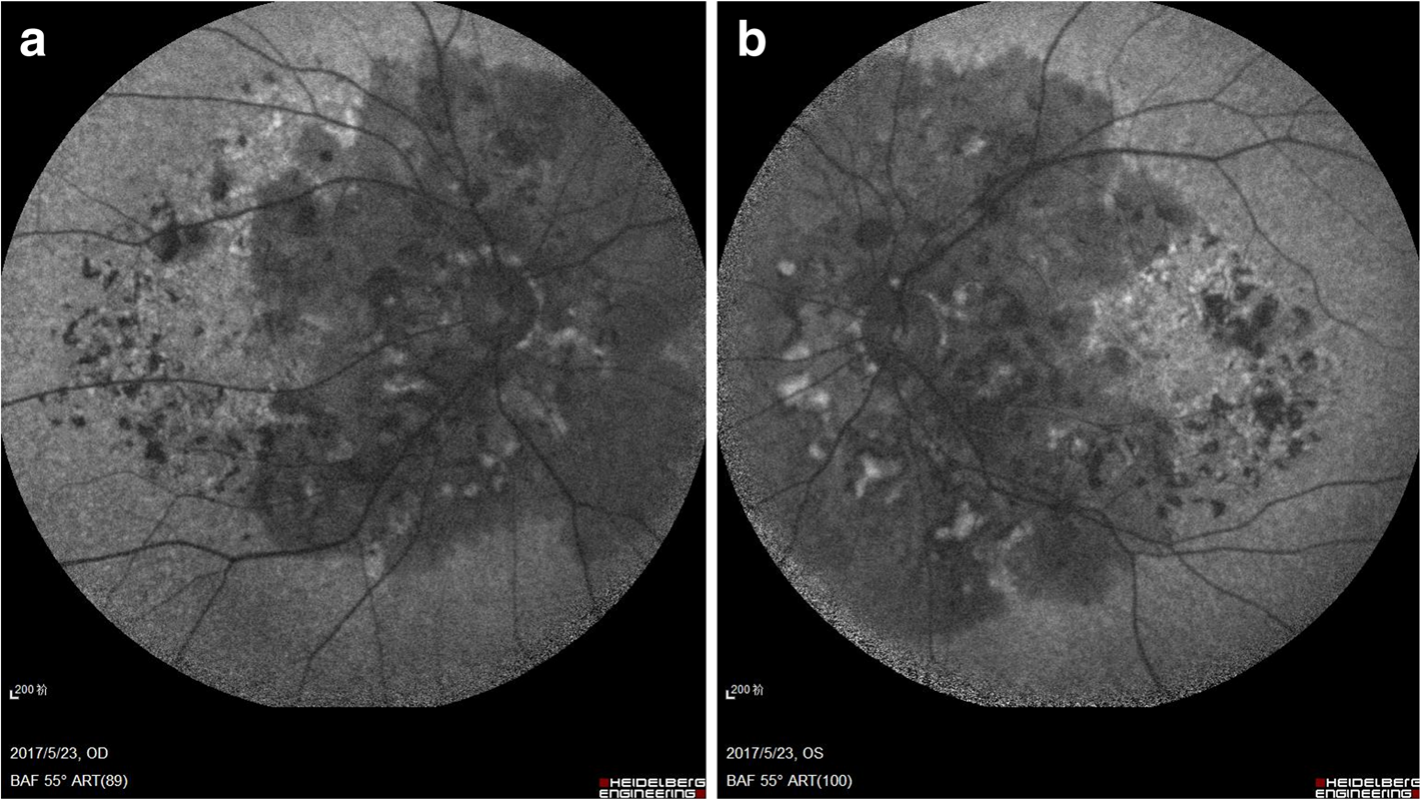
Fundus autofluorescence. Hypoautofluorescence was observed in the posterior fundus around the disc, which was corresponded to the surrounding black lesions of optic in fundus color photography (**a**: right eye; **b**: left eye)

**Fig. 3:**
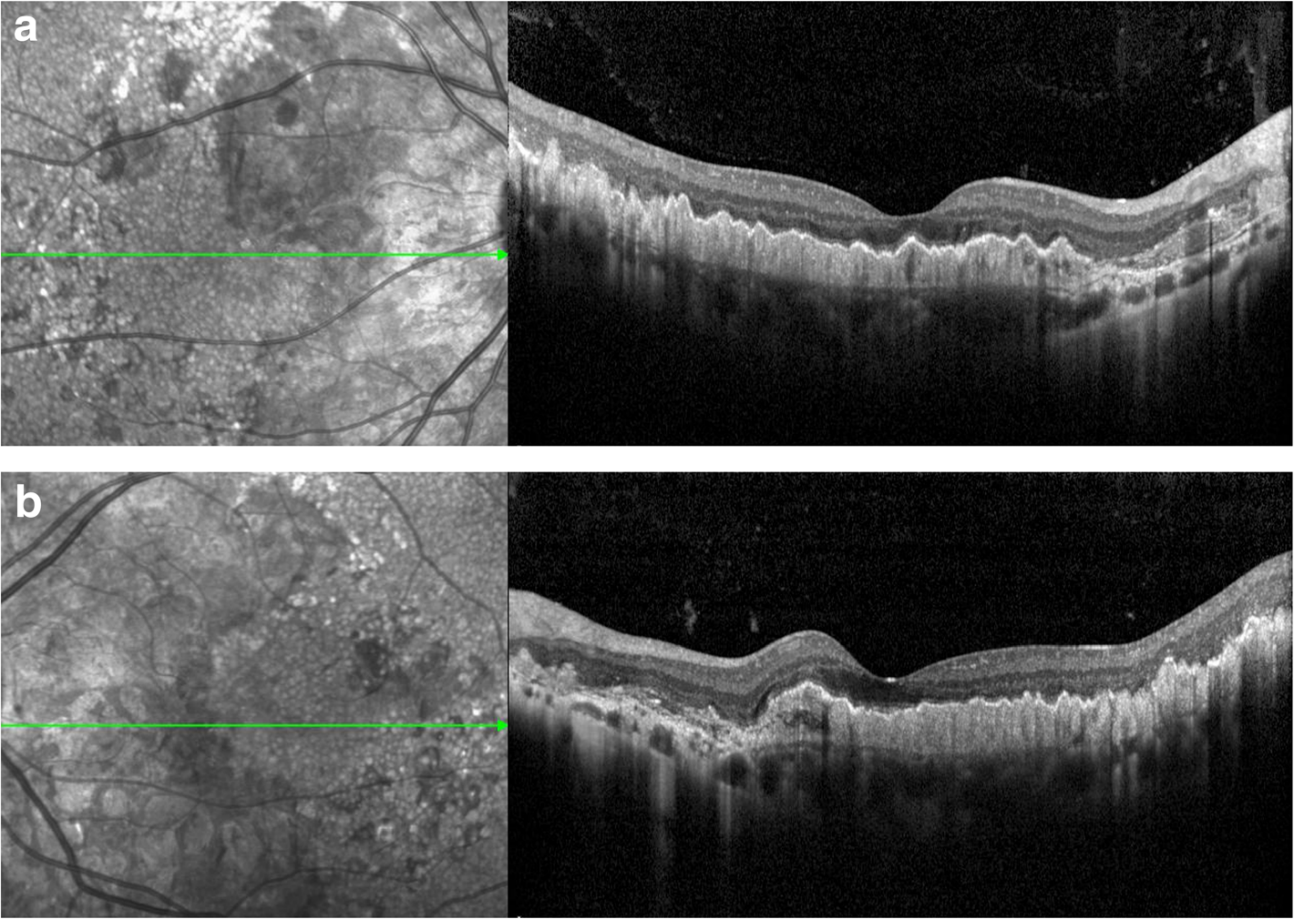
OCT scanning. A hyperreflective thickening was noticed beneath the RPE accompanied by wavy uplift (**a**: right eye; **b**:left eye)

**Fig. 4:**
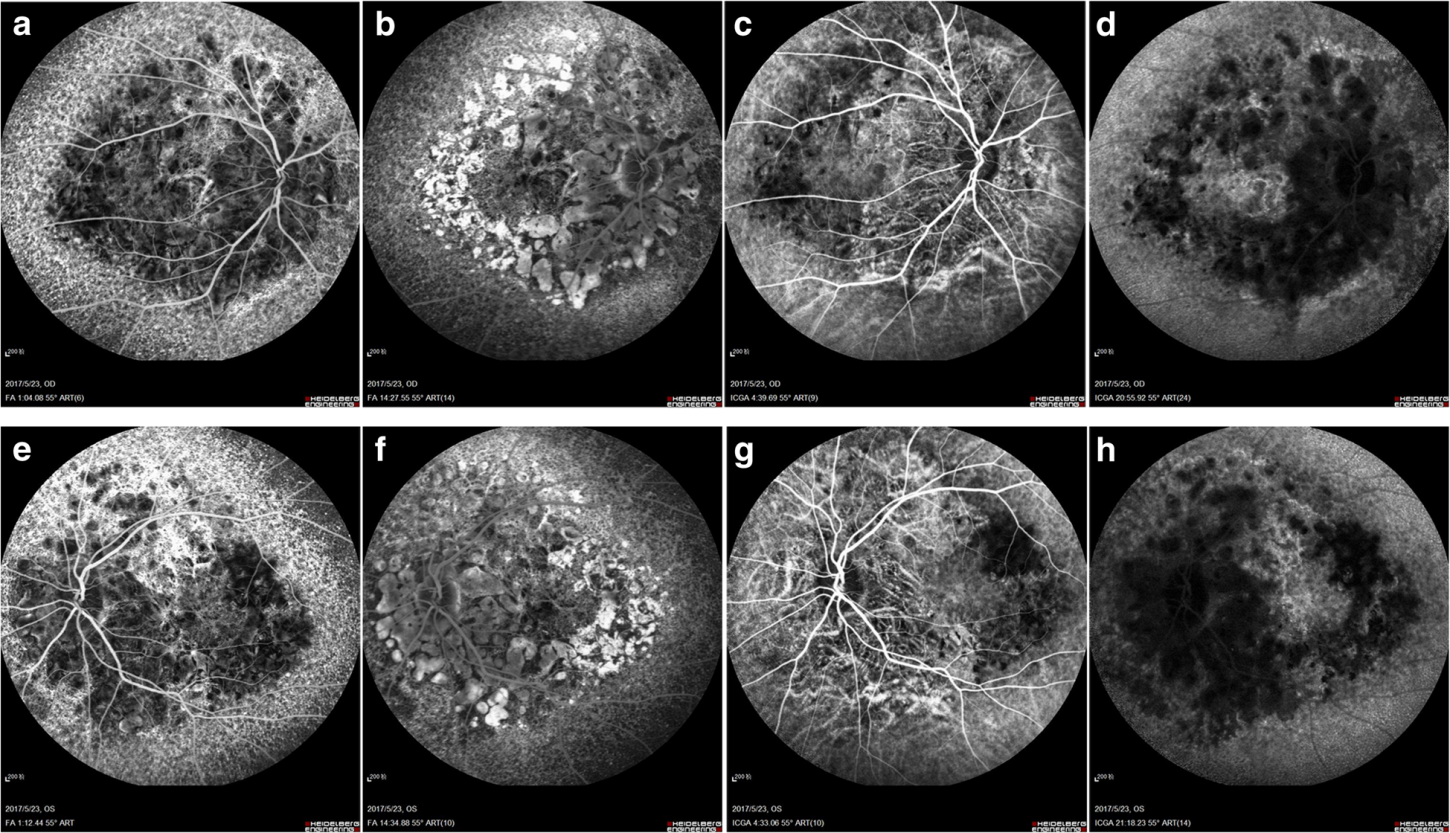
FFA and ICGA. In the early phase of FFA, the background fluorescence was observed around the disc decreased with choroidal vascular exposed, and diffuse pinpoint hyperfluorescent leakage was noticed in the peripheral parts reached to the equator (**a**: right eye; **e**: left eye). In the late phase of FFA, the hypofluorescence around the disc grew to hyperfluorescence formed to be “honeycomb” appearance in the posterior pole (**b**: right eye; **f**: left eye). In the early phase of ICGA, choroidal vascular exposed in the posterior pole around the disc (**c**: right eye; **g**: left eye). In the late phase of ICGA, massive hypofluorescence of choroidal was noticed in the posterior pole around the disc, while hyperfluorescence was observed in the peripheral parts reached to the equator (**d**:-right eye; **h**: left eye)

**Fig. 5:**
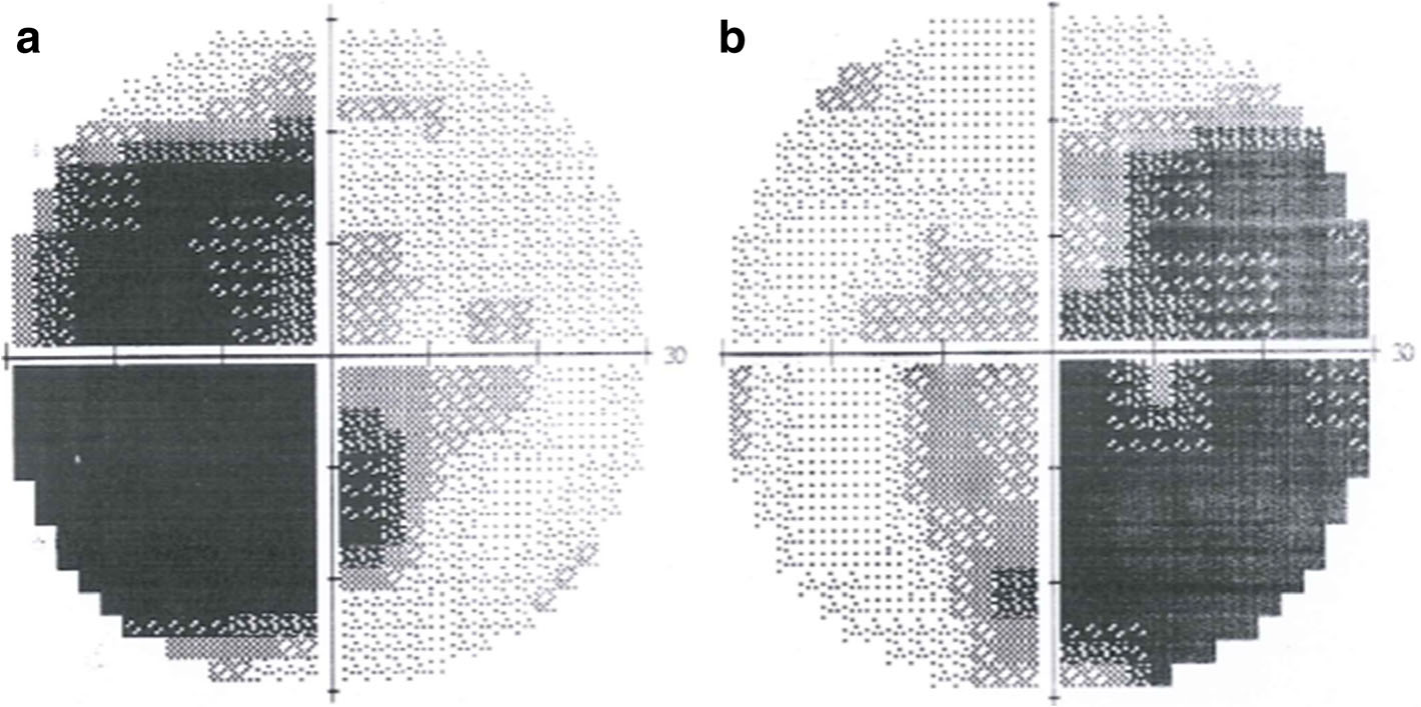
Humphery perimeter (central 30°). Visual field defect in the temporal sides of both eyes (**a**: left eye; **b**: right eye)

A 28-year-old female patient presented to our department due to blurry visual acuity for 10 years especially in the right eye with deterioration for 5 months. The best corrected visual acuity (BCVA) in the right and left eyes was 5.0 and 5.1 (logmar visual acuity chart), respectively. The intraocular pressure (IOP) in the right and left eyes was 17 mmHg and 18 mmHg, respectively. No abnormalities were observed in the anterior segment of both eyes. Fundus examination showed that the optic disc was normal, while the remarkable diffuse pinpoint or drusen-like speckle yellow white lesions affected the posterior fundus, with varying degrees of retina/retinal pigment epithelium (RPE)/choroid atrophy around the disc. The fovea light reflex was not clear (Figs. 1 and 2). Optical coherence tomography (OCT) scan showed extensive hyperreflective thickening beneath the retinal pigment epithelium (RPE, Fig. 3). Fundus fluorescein angiography (FFA) and indocyanine green angiography (ICGA) assumed that RPE/choroidal disorder was featured by “honeycomb” appearance (Fig. 4). Visual field showed defect in the temporal sides (Fig. 5). Electrophysiological examination findings (e.g. ERG, EOG, and VEP) were normal.

**Fig. 6:**
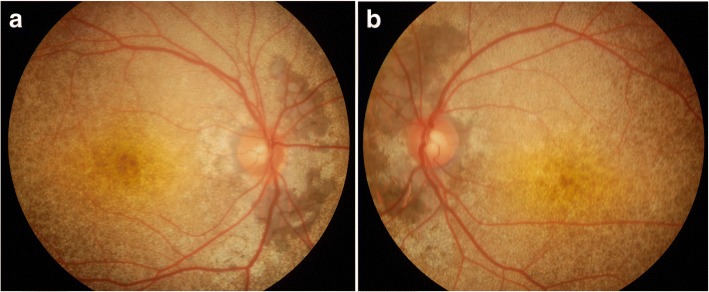
Fundus color photography of the brother. RPE/choroid atrophy was noticed in the posterior fundus around the disc, with diffuse pinpoint or speckle yellowish white lesions involving the peripheral area reached to the equator(**a**: right eye; **b**: left eye)

**Fig. 7:**
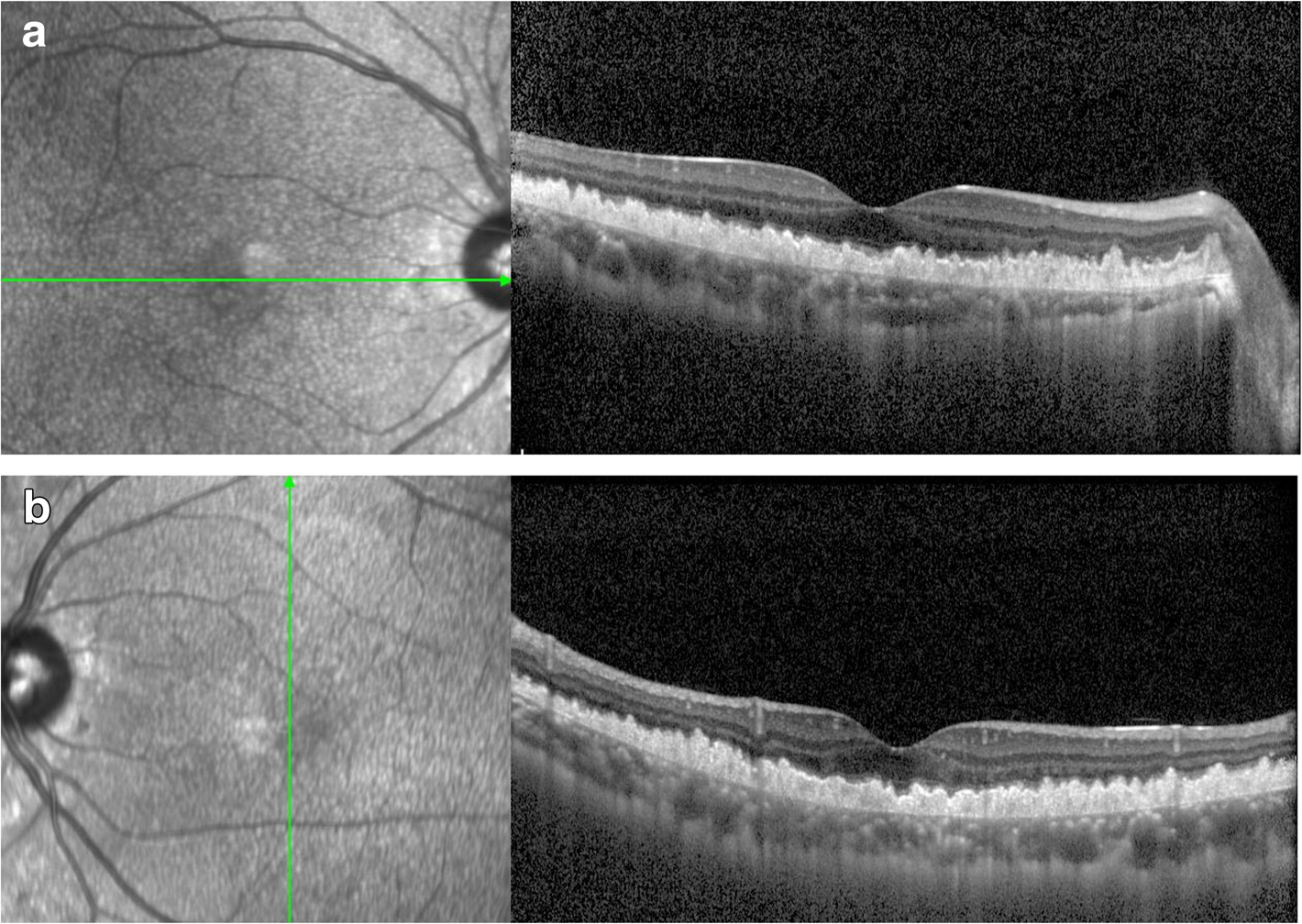
OCT scanning of the brother. A hyperreflective thickening was noticed beneath the RPE (**a**: right eye; **b**: left eye)

Her 22-year-old brother showed BCVA of 5.0 (logmar visual acuity chart) in both eyes. Fundus examination showed diffuse pinpoint yellow white deposits throughout the macular and peripapillar area with honeycomb-like pigmentary changes around the disc (Fig. 6a and b). OCT scan showed a hyperreflective thickening beneath the pigmentary epithelium accompanied by wavy uplift (Fig. 7a and b).

**Fig. 8:**
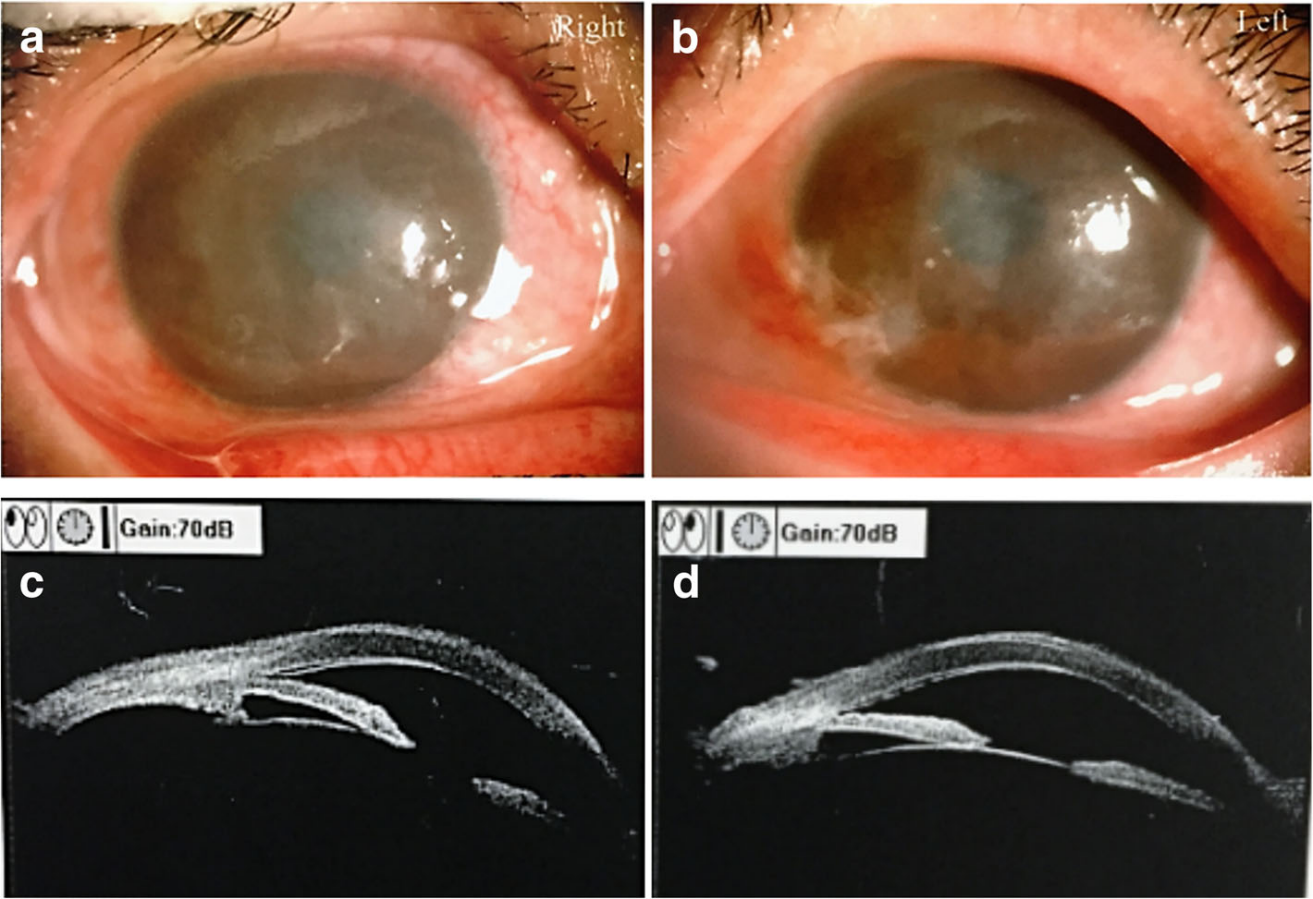
Corneal photography and OCT scan of the mother. Corneal opacity was noticed, and was uneven (**a**: right eye; **b**: left eye). The corneal epithelium was thicken and coarse, and detachment was noticed in the corneal endothelium at the 12 o’clock position(**c**: right eye; **d**: left eye)

Her 54-year-old mother complained of poor visual acuity for at least 20 years, especially at nighttime. The BCVA of the right eye was FC/10 cm, and the left eye was 4.0 (logmar visual acuity chart). She had corneal opacity in both eyes (Fig. 8a and b), and the fundus could not be observed clearly. Corneal scan of OCT showed granular cloudiness, and corneal endothelium detachment in the peripheral part (Fig. 8c and d). Color Doppler ultrasonography of eyeball showed thickening in the posterior wall of both eyeballs.

Her father showed no history of ocular diseases. He would not come to our hospital for the ocular examinations due to personal reasons.

**Fig. 9:**
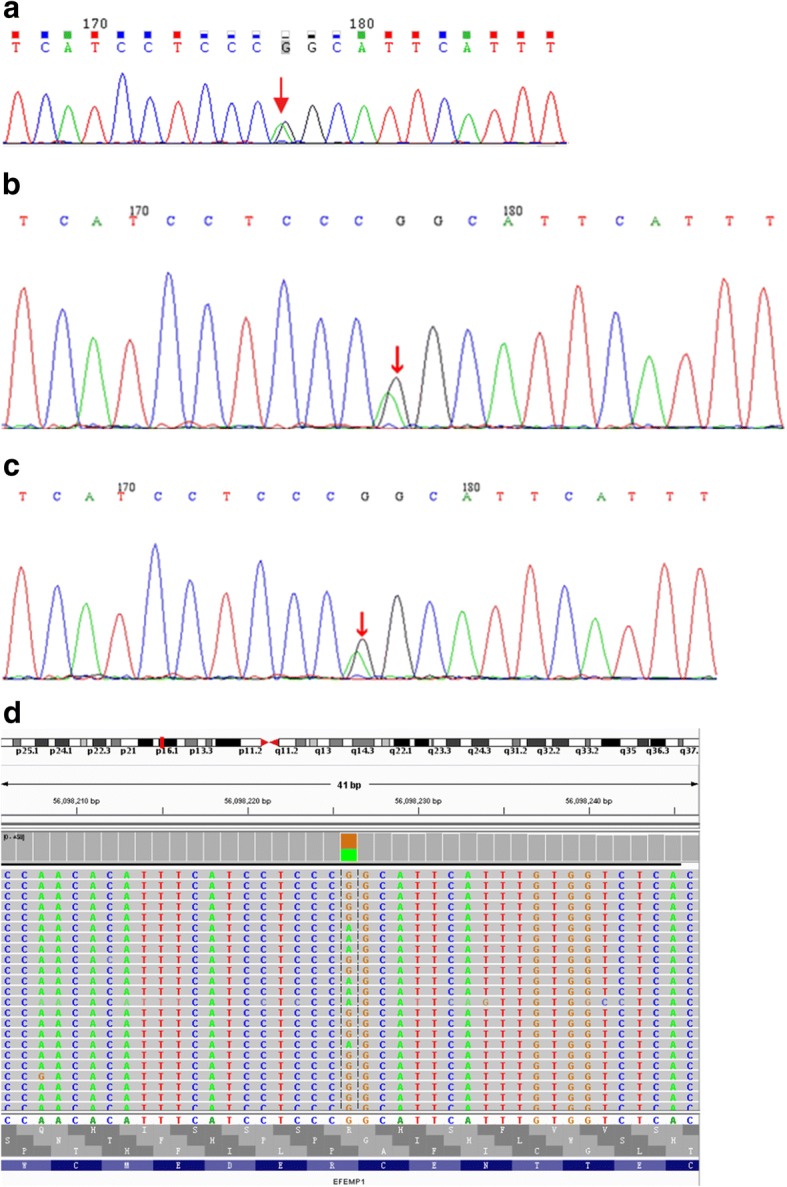
Gene sequencing results. The first generation sequencing results indicated mutation of EFEMP1 (c.1033C > T, p.R345 W) in the patient (**a**), his brother (**b**) and mother (**c**). The second generation sequencing results for the EFEMP1 gene (**d**). Sequencing findings for the patient

After signing the informed consents, venous blood was collected from the female patient, her mother and her brother, respectively. Gene sequencing was performed by MyGenostics (Peking, China). Gene sequencing revealed heterozygous mutations in *EFEMP1* gene, which was consistent with the DHRD/ML. The study protocols were approved by the Ethical Committee of Hainan General Hospital. For the gene sequencing, single heterozygous mutation (*c.1033C > T*) was observed in each of the three blood samples. This missense mutation triggered *p.R345W* (Fig. 9).

## Discussion

DHRD was initially reported in 1899 by Doyne, which was featured by closely-grouped white spots involving the disc-macular area in a pattern termed honeycomb [4]. Also, it is manifested as diffuse retina white spots. To our best knowledge, ML was initially described initially in inhabitants or descendants in the Leventine valley of Tessin Canton in Southern Switzerland [5]. In a previous study, Haimovici et al. [6] summarized the typical onset age and features of such condition, which was featured by drusen-like deposits in the macula and around the optic nerve that were usually apparent by the age of 20. Moreover, most of the patients were asymptomatic, and then the patients developed more numerous and larger drusen. In this literature, the authors also mentioned that the earliest visual symptoms included dyschromatopsia, metamorphopsia, or relative scotomas at the age of 30–40 yrs. Central visual acuity usually showed deterioration at the age of 40–50 yrs., however, it may occur at 30 or be delayed until 60 yrs [6]. In the later stages, the majority of patients showed loss of central vision and absolute scotomas, which was associated with the development of extensive pigmentary changes and geographic atrophy (GA) or choroidal neovascularization (CNV) in the region of the confluent macular drusen [6].

Recently, DHRD and ML are considered as the same disease because of similar clinical symptoms. In a previous study, Stone et al. revealed a single non-conservative mutation (Arg345Trp) in *EFEMP1* gene that was associated with the pathogenesis of ML and DHRD [1]. *EFEMP1* gene, localized on chromosome 2p16–21, encodes an extracellular matrix protein expressed in the retina, RPE and choroid [7]. Besides, it showed homology among the protein members [8]. *EFEMP1*-encoded protein may interact with the other extracellular matrix proteins such as connexin, collagen, fibronectin, and laminin, which contributed to the formation of drusen [1]. The clinical symptoms and pathogenesis of DHRD/ML are similar with that of age-related macular degeneration (AMD), which is featured by soft drusen, external RPE structure loss, RPE vacuolation and atrophy, as well as the final neovascularization at the age of less than 40 yrs. [9]. However, they are indeed different diseases. The mutation (Arg345Trp) of *EFEMP1* was reported to show no association with the drusen related lesions such as AMD [10]. Meanwhile, no Arg345Trp mutation was identified in the cases with dispersed or familial AMD [11]. Thus far, the Arg345Trp mutation is the only mutation identified in the coding or adjacent intronic regions of *EFEMP1* gene in familial or sporadic early onset drusen. This suggests that the Arg345Trp mutation is responsible for producing a specific drusen phenotype (drusen around the optic nerve head in DHRD and radial drusen in ML) [12]. In a previous study aimed to distinguish DHRD apart from ML, DHRD patients were considered to present comby drusen in the posterior fundus, while smaller discrete drusen radially distributed around the macula in ML patients [13].

To date, there were few reports of ML/DHRD. In China mainland, there was only one case report of R345W mutation in *EFEMP1* caused ML/DHRD in a family [14]. In that report, 6 members of one family at the age of 37–61 yrs., and the proband was 43 yrs., all of whom showed drusen distributed in the fundus, 2 had RPE disorders and CNV. In this study, we identified a heterozygous mutation of *EFEMP1* in a family with a 28-yrs sister of proband, a 22-yrs brother, and their mother. Our clinical findings were not similar with previously reported cases, despite some similarities [11–15]. Firstly, the young proband in this study presented clustered drusen reaching the equator of fundus with no CNV in the macula, while all previous cases showed drusen only localized in the macular region not exceeding the vascular arch. Secondly, besides remarkable drusen, there were large dark gray lesions originated from RPE/choroid atrophy in the posterior pole around the disc. No such feature was reported in the previous literature, even a few cases presented pigment proliferation in the macula [11–14]. Thirdly, the vision of the patient and her brother was not significantly impaired despite of the wide damages in the fundus. Some reports [4, 12] showed declined vision if there were densely large drusen in the macular area, which may deteriorate when combing with CNV and RPE disorder. However, in the 5-yr follow-up [16] of a Japanese female patient with DHRD/ML, one eye of BCVA remained normal in spite of aggravation of drusen and pigmentation in the macula, and the other eye showed vision loss due to CNV of macula. Furthermore, the brother present remarkable diffused drusen, while milder RPE /choroid damages of honeycomb-like appearance were noticed in the posterior pole around the disc. Nevertheless, it showed no clinical symptoms at present. The sister presented visual field defects corresponding to the peripapillary RPE/choroid damages. Thus, we deduced the pinpoint, yellowish, white drusen might represent the early stage of this disease, which then fused to patchy drusen. However, the dark gray lesions of RPE/choroid atrophy around the optic disc might imply progress of the disease with following vision loss. Multifocal ERG, OCT or FFA/ICGA examinations and visual field examinations may contribute to long-term follow-up [16–18].

For the treatment, Lenassi et al. [19] reported that low-energy laser treatment was safe and might be effective for treating autosomal dominant drusen induced by *EFEMP1* mutation. In 11 patients with autosomal dominant drusen and confirmed with disease-causing *EFEMP1* mutation, the eyes with poor vision were treated with Argon green laser. The untreated eyes lost an average of 0.8 letters, whereas, the treated eyes gained an average of 4.9 letters. The average thickness of the drusen in the untreated eyes showed increase, while no changes were noticed in the treated eyes. Meanwhile, Sohn et al. [20] showed that CNV in DHRD was sensitive to treatment with intravitreal bevacizumab which was featured by absorption of subretinal fluid and improvement of visual acuity in two cases.

In this study, the patient and her brother had almost normal central vision, which might the associated with the fact that the lesion did not involve the fovea. We implied that there would be a vision loss when the RPE/choroid atrophy invaded the macula. Anyway, long-term follow up was still required, and immediate treatment was recommended in cases of deterioration of drusen or CNV formation, in order to keep the vision. On the other hand, her mother showed long-term corneal opacity with granular cloudiness. Up to now, we are not sure whether these lesions are associated with the DHRD/ML. In the future, we hope to find out the exact relations between them.

## Abbreviations

AMD: Age-related macular degeneration
BCVA: Best corrected visual acuity
CNV: Choroidal neovascularization
DHRD: Doyne honeycomb retinal dystroph
FFA: Fundus fluorescein angiography
GA: Geographic atrophy
ICGA: Indocyanine green angiography
IOP: Intraocular pressure
ML: Malattia leventinese
OCT: Optical coherence tomography
RPE: Retinal pigment epithelium
